# The Cholinergic Amelioration of Sepsis-Induced Baroreflex Dysfunction and Brainstem Inflammation Is Negated by Central Adenosine A3 Receptors

**DOI:** 10.3390/ph18030388

**Published:** 2025-03-09

**Authors:** Amany E. El-Naggar, Mai M. Helmy, Sahar M. El-Gowilly, Mahmoud M. El-Mas

**Affiliations:** 1 Department of Pharmacology and Toxicology, Faculty of Pharmacy, Alexandria University, Alexandria 21511, Egypt; amany.elnaggar@alexu.edu.eg (A.E.E.-N.); mai.helmy@alexu.edu.eg (M.M.H.); sahar.elgowilly@alexu.edu.eg (S.M.E.-G.); 2 Department of Pharmacology and Toxicology, Faculty of Medicine, College of Medicine, Kuwait University, Jabriya 46301, Kuwait

**Keywords:** sepsis, reflex bradycardia, nicotine, adenosine A3 receptors, neuroinflammation, nucleus tractus solitarius

## Abstract

**Background/Objectives**: Sepsis has been shown to depress arterial baroreceptor function, and this effect is counterbalanced by the cholinergic anti-inflammatory pathway. Considering the importance of central adenosine receptors in baroreceptor function, this study tested whether central adenosine A3 receptors (A3ARs) modulate the cholinergic-baroreflex interaction in sepsis and whether this interaction is modulated by mitogen-activated protein kinases (MAPKs) and related proinflammatory cytokines. **Methods**: Sepsis was induced by cecal ligation and puncture (CLP) and rats were instrumented with femoral and intracisternal (i.c.) catheters. Baroreflex sensitivity (BRS) was measured 24 h later in conscious animals using the vasoactive method, which correlates changes in blood pressure caused by i.v. phenylephrine (PE) and sodium nitroprusside (SNP) to concomitant reciprocal changes in heart rate. **Results**: The reduction in reflex bradycardic (BRS-PE), but not tachycardic (BRS-SNP), responses elicited by CLP was reversed by i.v. nicotine in a dose-related manner. The BRS-PE effect of nicotine was blunted following intracisternal administration of IB-MECA (A3AR agonist, 4 µg/rat). The depressant action of IB-MECA on the BRS facilitatory action of nicotine was abrogated following central inhibition of MAPK-JNK (SP 600125), PI3K (wortmannin), and TNFα (infliximab), but not MAPK-ERK (PD 98059). Additionally, the nicotine suppression of sepsis-induced upregulation of NFκB and NOX2 expression in the nucleus tractus solitarius (NTS) was negated by A3AR activation. The molecular effect of IB-MECA on NFκB expression disappeared in the presence of SP 600125, wortmannin, or infliximab. **Conclusions**: The central PI3K/MAPK-JNK/TNFα pathway contributes to the restraining action of A3ARs on cholinergic amelioration of sepsis-induced central neuroinflammatory responses and impairment of the baroreceptor-mediated negative chronotropism.

## 1. Introduction

Hypotension and myocardial dysfunction are common after-effects of sepsis [1,2,3]. The precise mechanisms of septic cardiomyopathy are not clear, but contributing factors include depression of β-adrenergic signaling, mitochondrial dysfunction, inflammation, and oxidative stress [4,5]. The suppression of baroreceptor function correlates with septic autonomic cardiomyopathy and accelerated mortality in experimental [6,7,8] and clinical settings [6,9]. Along with the depressed baroreflex-mediated control of heart rate (HR) [6,7,8], the attenuation of central control of renal and splanchnic sympathetic activities is also blamed on sepsis [10,11].

The cholinergic anti-inflammatory system acts to counteract irregularities in the immune response associated with inflammatory conditions like neurodegenerative diseases, multiple sclerosis, and sepsis [12]. Our recent studies showed that cholinergic activation by nicotine weakens septic cardiovascular sequalae [13,14] and associated depression of baroreflex-mediated HR responses through the activation of α7 and α4β2 nicotinic acetylcholine receptors (nAChR) [8,15]. The notion that nicotine effectively nullifies the inflammatory reaction prompted by a variety of septic challenges has been stressed [16,17,18].

The nucleoside adenosine plays a key role in the control of various physiological functions, including the immune response [19]. Contradictory reports are available regarding the role of peripheral A1ARs and A3AR3 in sepsis mortality and end-organ damage, with both positive [20,21] and negative effects [22,23] being reported. Specifically, the role of A3ARs in inflammation seems to be contradictory as both proinflammatory and anti-inflammatory actions have been reported [19]. There is also evidence for a paradoxical function for A3ARs in the development and progression of sepsis, although the number of studies reported in this regard is limited. Lee et al. [21] demonstrated that A3AR activation in mice with septic peritonitis guards against renal and hepatic injuries and associated hyperacute inflammatory response [21]. By contrast, central A3ARs aggravate septic manifestation of hypotension and autonomic neuropathy [14,24,25,26]. Others failed to validate any role for A3ARs in microglial activation evoked by systemic lipopolysaccharides in mice [27]. It is possible, therefore, that the inflammatory effect of A3AR receptors during sepsis depends largely on the type of septic injury and tissue damage.

That being said, no studies are available on whether the depressed baroreceptor function in sepsis could be modulated via central adenosine receptors. Scislo et al. [28] reported that A1ARs of the NTS, the main integrating site of baroreceptor inputs [29], inhibit sympathetic reflexes to the unloading of arterial baroreceptors. Scislo and O’Leary [30] also reported that A2aARs augment baroreceptor activity by facilitating the brainstem glutamatergic transmission. Unlike A1ARs and A2aARs, little or no information is available about the role of A3ARs in baroreflex control. The primary objective of this study was to test the hypotheses that (i) the depressed arterial baroreceptor function in septic rats and its counteraction by nicotine are modulated by central A3ARs, and (ii) central PI3K/MAPKs and associated inflammatory (NFκB/TNFα) and oxidative (NOX2) signals contribute to the nicotine-A3AR interaction. Sepsis was induced by cecal ligation and puncture 24 h before baroreflex measurement and baroreflex sensitivity (BRS) was measured using the vasoactive method. The cecal ligation and puncture model is considered the gold standard model for sepsis, with the advantage of high relevance to human sepsis [31,32].

## 2. Results

### 2.1. Nicotine Ameliorates the Baroreflex Dysfunction in Septic Rats

**Figure 1:**
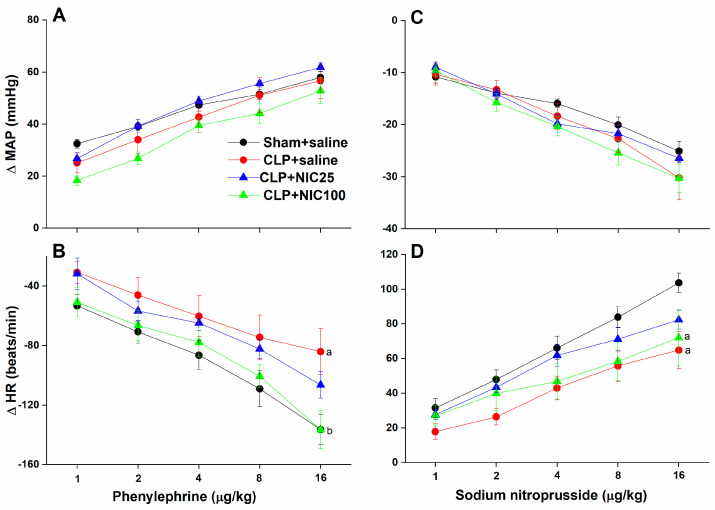
Effect of i.v. nicotine (NIC, 25−100 μg/kg) on increases and decreases in MAP (**A,C**) and associated reflex chronotropic responses (**B,D**) evoked by phenylephrine (1−16 μg/kg) and sodium nitroprusside (1−16 μg/kg), respectively, in septic rats. Values are means ± SEM of 7−8 observations. ^a^ *p* < 0.05 vs. “sham/saline” values, ^b^ *p* < 0.05 vs. “CLP/saline” values.

**Figure 2:**
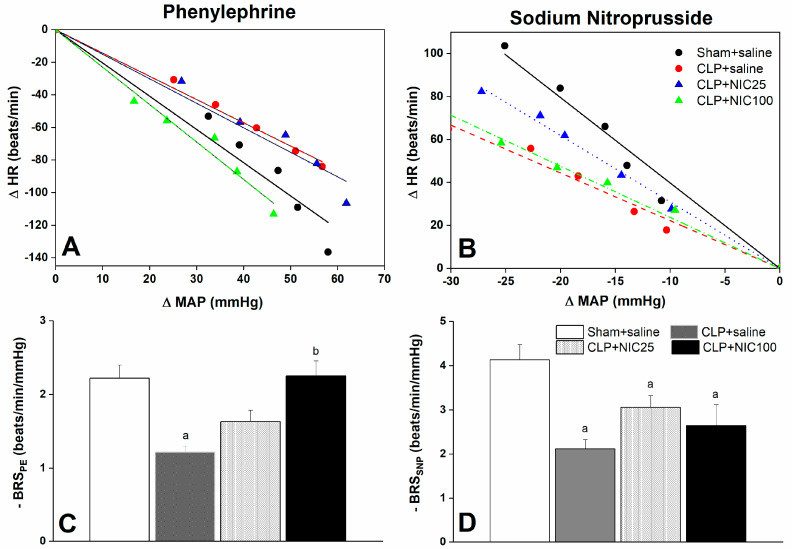
Effect of i.v. nicotine (NIC, 25−100 μg/kg) on baroreflex curves generated by phenylephrine (PE, 1−16 μg/kg) or sodium nitroprusside (SNP, 1−16 μg/kg) (**A**,**B**) and slopes of regression lines (**C**,**D**) in septic rats. Values are means ± SEM of 7−8 observations. ^a^ *p* < 0.05 vs. “sham/saline” values, ^b^ *p* < 0.05 vs. “CLP/saline” values.

The effects of nicotine (25 and 100 μg/kg i.v.) on baroreflex responses measured by the vasoactive method in conscious CLP rats are depicted in Figure 1 and Figure 2. In all groups, the i.v. administration of serial dilutions of PE and SNP (1–16 μg/kg each) elicited dose-dependent increases and decreases in MAP, respectively, which were paralleled with reciprocal changes in HR (Figure 1). CLP had no effect on changes in MAP caused by PE (Figure 1A) or SNP (Figure 1C), but significantly reduced the respective baroreceptor-mediated falls (Figure 1B) and rises (Figure 1D) in HR. The examination of the baroreflex curves, which correlate changes in HR to associated changes in MAP, revealed that CLP caused upward and downward shifts in the baroreflex curves constructed by PE or SNP, respectively (Figure 2A,B), and significantly reduced the slopes of these curves, BRS_PE_ and BRS_SNP_, by about 50% compared with sham values (Figure 2C,D). The CLP-evoked upward shifts in PE baroreflex curves and associated reduction in BRS_PE_ were dose-dependently alleviated by i.v. administration of nicotine (25 and 100 μg/kg) (Figure 2A–C). On the other hand, downward shifts in the baroreflex curves generated by SNP and concurrent reductions in BRS_SNP_ were not significantly altered by either dose of nicotine (Figure 2B–D).

### 2.2. The Favorable Baroreflex Action of Nicotine in Septic Rats Is Opposed by Central A3ARs

**Figure 3:**
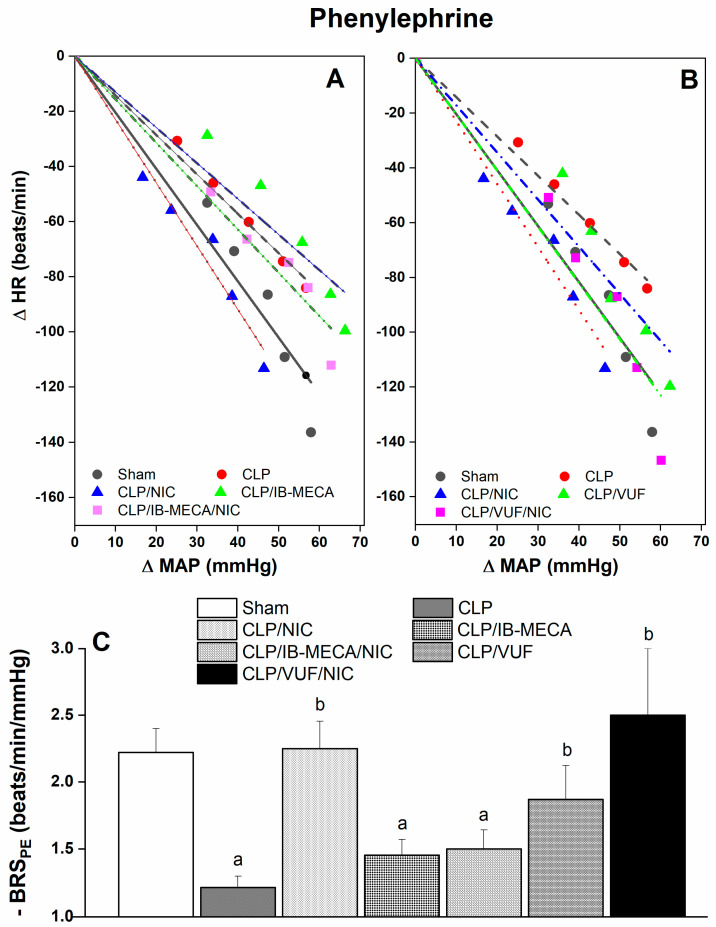
Effect of i.c. IB-MECA (A3AR agonist, 4 µg/rat) or VUF5574 (A3AR antagonist, 2 µg/rat) in the absence and presence of nicotine (NIC, 100 μg/kg) on baroreflex curves generated by phenylephrine (PE, 1−16 μg/kg) (**A**,**B**) and slopes of regression lines (**C**) in septic rats. Values are means ± SEM of 7−8 observations. ^a^ *p* < 0.05 vs. “sham/saline” values, ^b^ *p* < 0.05 vs. “CLP/saline” values.

The effects of activation or blockade of central A3ARs by i.c. IB-MECA and VUF5574, respectively, on reflex bradycardic responses in CLP rats treated with or without nicotine are shown in Figure 3. Downward shifts in PE baroreflex curves caused by CLP and associated reductions in BRS_PE_ were preserved after central activation of A3ARs by IB-MECA (4 µg/rat i.c, Figure 3A–C) but significantly improved after A3AR blockade by VUF5574 (2 µg/rat i.c., Figure 3B,C), suggesting a tonic restraining influence for central A3ARs on reflex bradycardia. This is further supported by the observation that the alleviating effect of systemic nicotine (100 μg/kg) on downward shifts in PE baroreflex curves (Figure 3A) and decreases in BRS_PE_ (Figure 3C) in CLP rats disappeared upon simultaneous activation of A3ARs by i.c. IB-MECA.

**Figure 4:**
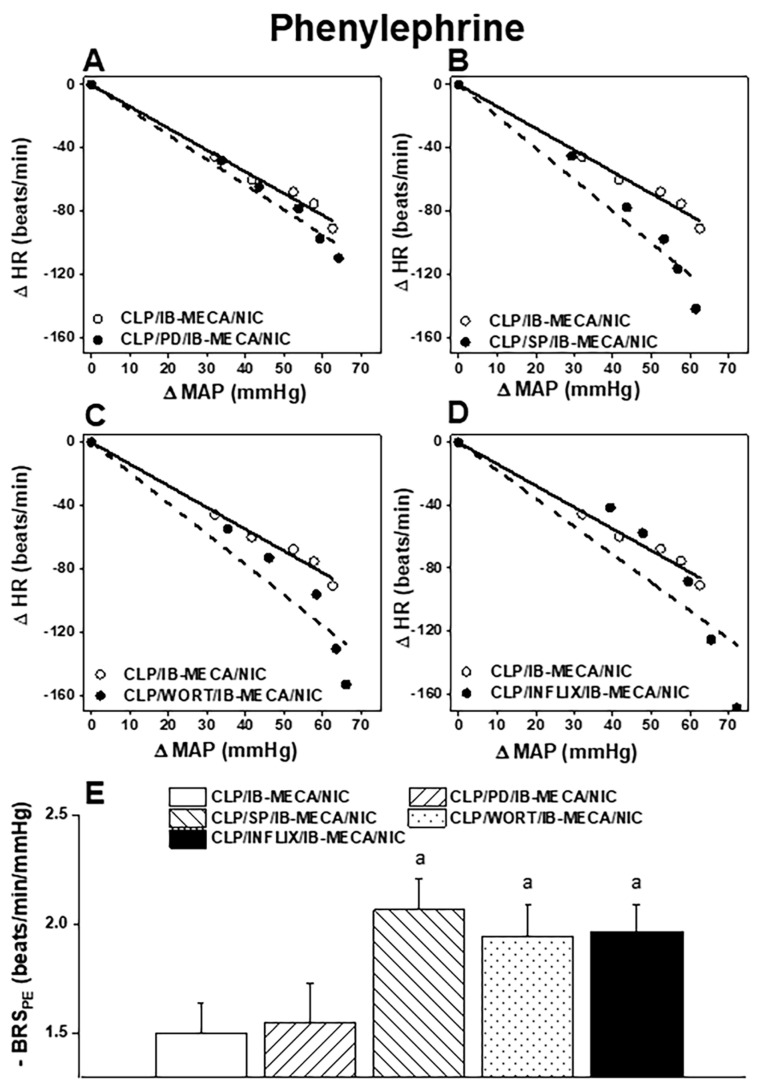
Effect of i.c. PD 98059 (MAPK-ERK inhibitor), SP 600125 (MAPK-JNK inhibitor), wortmannin (WORT, PI3K inhibitor), or infliximab (INFLIX, TNFα inhibitor) on baroreflex curves generated by phenylephrine (PE, 1−16 μg/kg, (**A**–**D**)) and slopes of the regression lines (**E**) in the presence of IB-MECA/nicotine-treated septic male rats. Values are means ± SEM of 7−8 observations. ^a^ *p* < 0.05 vs. “CLP/IB-MECA/NIC” values.

The possibility that central PI3K/MAPKs/TNFα signaling mediates the A3AR-nicotinic baroreflex interaction in sepsis was investigated. Figure 4 shows that the depressed BRS_PE_ (Figure 4E) and concomitant upward shifts in the PE baroreflex curves (Figure 4B–D) caused by IB-MECA in nicotine-treated septic rats were blunted after i.c. administration of SP 600125 (MAPK-JNK inhibitor, 30 µg/rat, i.c.), wortmannin (PI3K inhibitor, 0.5 µg/rat, i.c.), or infliximab (TNF-α inhibitor, 100 µg/rat, i.c.). By contrast, none of the above effects of IB-MECA was altered after i.c. inhibition of MAPK-ERK by PD 98059 (10 µg/rat, i.c.) (Figure 4A–E). Accordingly, the opposing role of IB-MECA in the nicotine-mediated amelioration of baroreflex dysfunction is mediated by PI3K/MAPK-JNK/TNFα pathways in septic rats.

### 2.3. NFκB and NOX2 Expressions in the Solitary Tract

**Figure 5:**
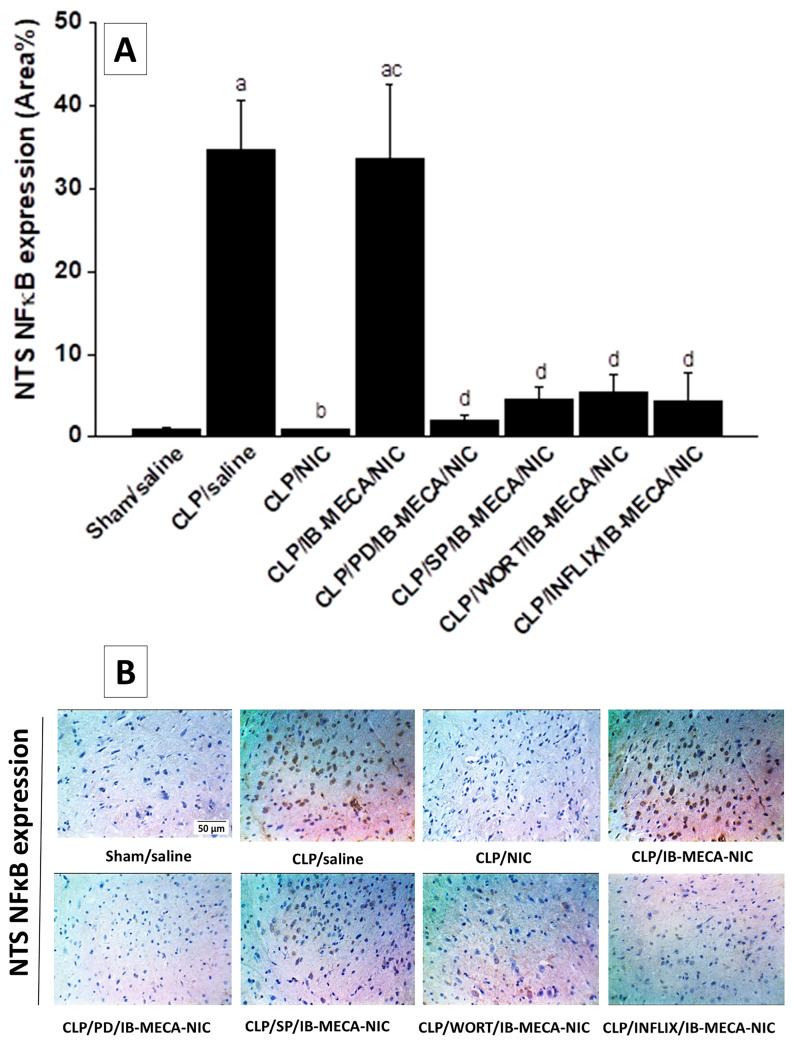
Effect of i.c. IB-MECA (A3AR agonist) alone and in the presence of PD 98059 (MAPK-ERK inhibitor), SP 600125 (MAPK-JNK inhibitor), wortmannin (WORT, PI3K inhibitor), or infliximab (INFLIX, TNFα inhibitor) on the nicotine-evoked downregulation of NFκB expression in septic (CLP) brainstem areas of NTS (A). Representative images are shown in panel B. ^a^ *p* < 0.05 vs. “sham/saline”, ^b^ *p* < 0.05 vs. “CLP/saline”, ^c^ *p* < 0.05 vs. “CLP/NIC”. ^d^ *p* < 0.05 vs. “CLP/IB-MECA/NIC”.

**Figure 6:**
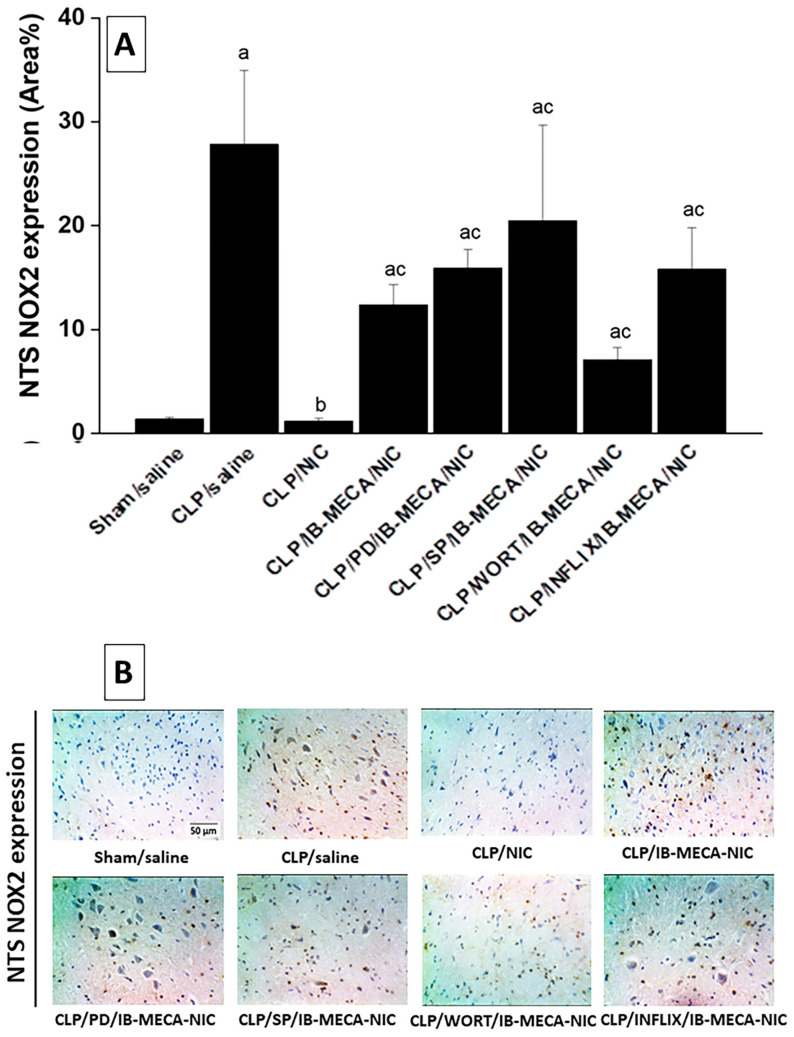
Effect of i.c. IB-MECA (A3AR agonist) alone and in the presence of PD 98059 (MAPK-ERK inhibitor), SP 600125 (MAPK-JNK inhibitor), wortmannin (WORT, PI3K inhibitor), or infliximab (INFLIX, TNFα inhibitor) on the nicotine-evoked downregulation of elevated NOX2 expression in septic (CLP) brainstem areas of NTS (**A**). Representative images are shown in panel (**B**). ^a^ *p* < 0.05 vs. “sham/saline”, ^b^ *p* < 0.05 vs. “CLP/saline”, ^c^ *p* < 0.05 vs. “CLP/NIC”.

Immunohistochemical staining showed that CLP caused significant elevations in the expression of inflammatory (NFκB, Figure 5A) and oxidative (NOX2, Figure 6A) signals in the brainstem neuronal pools of the NTS. The overexpressed signals of NFκB and NOX2 in septic brainstems were completely eliminated after treatment of CLP rats with nicotine (100 µg/kg, i.v.) and reappeared upon simultaneous i.c. administration of the A3AR agonist, IB-MECA. Further, the offsetting action of IB-MECA on the nicotine downregulation of NFκB expression in NTS areas of septic brainstems was counteracted by pharmacologic inhibition of central MAPK-ERK, MAPK-JNK, PI3K, or TNFα by i.c. PD 98059, SP 600125, wortmannin, and infliximab, respectively (Figure 5A). On the other hand, none of the abovementioned inhibitors affected the counteracting action of IB-MECA on nicotine-mediated inhibition of NOX2 expression in septic brainstems (Figure 6A). Representative images showing NTS expressions of NFκB and NOX2 are shown in Figure 5B and Figure 6B, respectively.

## 3. Discussion

This study is the first to report on the role of central A3ARs in the cholinergically mediated amelioration of baroreflex dysfunction in sepsis. The assessment of arterial baroreceptor activity using the vasoactive method revealed three important observations. First, while systemic nicotine reversed the attenuation caused by sepsis in baroreceptor-mediated falls in chronotropic responses, it failed to improve the simultaneous impairment in reflex rises in chronotropic activity. Second, the activation of central A3ARs by IB-MECA counterbalanced the boosting nicotine effect on reflex bradycardia, perhaps via the activation of the central PI3K/MAPK-JNK/TNFα cascade. Third, the upregulation of the proinflammatory cytokine NFκB in neural circuits of the brainstem solitary tract played a pivotal role in the nicotine/IB-MECA baroreflex interaction.

Arterial baroreceptors are stretch receptors that are primarily located in the aortic arch and carotid sinus and function to buffer abrupt fluctuations in blood pressure by controlling central sympathetic and vagal outflows [33]. The relative contributions of the two components of the autonomic nervous system to reflex chronotropic changes depend on the type of HR response, bradycardia or tachycardia. While reflex bradycardia is mediated mainly through the activation of cardiac parasympathetic nerves, reflex tachycardia follows the rise in cardiac sympathetic activity [33,34]. This study reports on the effect of sepsis on baroreceptor function and its interaction with nicotine. Consistent with previous reports [7,35], reflex bradycardic and tachycardic responses to PE and SNP, respectively, were both reduced when measured 24 h following CLP compared with sham operation. In addition to the impairment of baroreflex HR control, evidence from clinical and experimental studies showed that sepsis also inhibits baroreflex control of sympathetic discharges to non-cardiac tissues such as renal, splanchnic, and muscular sympathetic beds [10,11]. It should be noted that impaired baroreflexes are predictive of poor outcomes and reduced survival times during sepsis [6,9]. We also report that the depressant effect of sepsis on PE-mediated reflex bradycardic responses was dose-dependently alleviated by nicotine (25 and 100 μg/kg), with a significant increase in BRS_PE_ observed with the higher nicotine dose. This facilitatory action of nicotine on reflex bradycardia appears to be specific to the septic state because the same doses of nicotine had no effect on reflex decreases in heart rate when tested in intact, non-septic rats [36]. The lack of effect of nicotine on reflex tachycardic responses to SNP suggests a preferential reinforcing effect of nicotine on reflex cardiotonic vagal activity.

Despite the abundance of adenosine receptors and uptake sites in the central nervous system [29,37,38,39], and their diverse roles in the inflammatory response to sepsis and associated organ dysfunction [20,21,40,41,42,43], no studies are available on whether central adenosinergic pathways are involved in baroreflex dysfunction induced by sepsis. Therefore, the primary goal of this study was to determine whether the depressed arterial baroreceptor function in septic rats and its counteraction by nicotine are modulated by central A3ARs. Current pharmacological studies provided two observations that infer a provocative role for central A3ARs on baroreflex dysfunction induced by sepsis. First, like the effect of nicotine, the selective blockade of central A3ARs by central administration of VUF5574 into the cisterna magnum reversed the sepsis-evoked impairment of reflex bradycardic responses and restored BRS_PE_ to near-sham-operated values. The second observation relates to the finding that activation of central A3ARs by i.c. IB-MECA abolished the enhancing effect of nicotine on reflex bradycardia in septic rats. The data obtained from agonistic and antagonistic studies reveal a hostile role for central A3ARs in the worsened vagal baroreceptor activity in septic rats. A similar outcome has been reported in the genetic study reported by Inoue et al. [23], in which less lung injury and improved survivability are demonstrated in A3AR-knockout CLP mice compared with wild-type septic animals. It is imperative to comment here on the relationship between baroreflex function and brainstem inflammation. A number of studies have demonstrated the positive relationship between NTS neuroinflammation and baroreflex dysfunction [44,45]. Bearing this in mind, it is conceivable that A3AR-induced worsening of baroreflex function in sepsis might be related to upregulation of the central NFκB inflammatory signals.

Immunohistochemical studies were pursued in this investigation to determine the roles of inflammatory (NFκB) and oxidative (NOX2) entities of the brainstem solitary tract in the nicotine/A3AR interaction. Whereas NFκB is a key proinflammatory cytokine that promotes the production of downstream inflammatory signals, NOX2 is an enzyme that generates reactive oxygen species that trigger septic oxidative damage [46,47]. We particularly targeted the NTS because of its crucial role in baroreflex control [48,49] and in the neuromodulation of the immune responses [15,50,51]. Our data showed that the attenuated reflex bradycardia in CLP rats was coupled with upregulated expression of NFκB and NOX2 in the NTS compared with the respective sham values. These findings are consistent with reported studies on the positive correlation between baroreflex dysfunction and neuroinflammation [44,45]. More importantly, we also showed that the heightened NTS expression of NFκB and NOX2 was restored to near-sham values after nicotine administration and fully (NFκB) or partially (NOX2) reinstated upon central treatment with the A3AR agonist IB-MECA. Together, these results implicate neuroinflammatory and neuro-oxidative machineries of the solitary tract in the disruptive action of central A3ARs on the cholinergic defense against baroreflex dysfunction induced by sepsis.

Signal transduction studies showed that the activation of TLR4, an essential component of the innate immune system, by sepsis causes downstream enhancement of PI3K/MAPK signaling, nuclear translocation of NFκB, and subsequent generation of proinflammatory cytokines such as TNFα and interleukins [52]. Given the established roles of these inflammatory signals in baroreflex modulation in septic [53,54] and non-septic states [55,56,57,58,59], we investigated the possibility that the inhibitory effect of A3AR agonism on the privileged baroreflex and neuroinflammatory action of nicotine would be eliminated after pharmacologic targeting of individual components of the central PI3K/MAPK/TNFα cascade. We found that central inhibition of PI3K (wortmannin), MAPK-JNK (SP 600125), or TNFα (infliximab) restored the baroreflex enhancing effect of nicotine in IB-MECA-pretreated endotoxic rats. This seemingly favorable baroreflex action coincided with a depressed expression of the solitary tract NFκB, but not NOX2, inferring a preferential involvement of the inflammatory NFκB in the PI3K/MAPK-dependent IB-MECA/nicotine baroreflex interaction in sepsis. Considering the contradictory roles of the four isoforms of NOX in sepsis pathophysiology [60,61,62], more studies appear to be needed to assess the roles of other NOX isoforms in the A3AR/nicotinic interaction in sepsis-related baroreflex dysfunction.

Our finding that the central inhibition of MAPK-JNK (SP 600125), but not MAPK-ERK (PD98059), counteracted the depressant action of IB-MECA on the baroreflex facilitatory effect of nicotine and restored baroreflex function to near-physiologic levels suggests a differential contribution of the two MAPK isoforms to the baroreflex nicotine/IB-MECA interaction in sepsis. Our data are consistent with earlier reports that suggested that the recruitment of individual MAPKs depends on the nature of the cardiovascular response to sepsis. For example, Sallam et al. showed that while central MAPK-JNK is required for sepsis-induced hypotension but not autonomic dysfunction, MAPK-ERK is not involved in either response [63,64]. In another study, central MAPK-JNK and MAPK-ERK are implicated in the amelioration by the immunosuppressant drug cyclosporine of cardiac autonomic neuropathy induced by sepsis, but only MAPK-ERK seems to provoke the cyclosporine reversal of the associated decline in blood pressure [63,64]. The variable roles of MAPKs in other sepsis-unrelated disorders like hypoxia and osteoarthritic have also been noted [65,66].

It is important to comment on three possible limitations of this study. First, in addition to the anti-inflammatory action of nicotine mediated mainly via activation of α7- nAChRs, nicotine can also activate a diversity of other nAChRs, such as α1 and α4β2-nAChR [17,67,68,69,70,71,72], as well as non-nicotinic receptors and channels [73,74,75,76,77]. Similarly, the A3AR agonist IB-MECA is believed to activate, with lower affinity, A2aAR, which contributes to its antioxidative and cardioprotective actions [78,79]. The potential involvement of these off-target effects of nicotine and IB-MECA in current findings cannot be overlooked and requires further investigations. Second, we also acknowledge the limitation of not studying the effects of individual inhibitors of the PI3K/MAPKs/TNFα signaling in the absence of nicotine on measured cardiovascular functions. Considering our current finding that the A3AR agonist IB-MECA counteracted the favorable nicotine action on baroreflex dysfunction in septic rats, we only determined the effect of these inhibitors on the IB-MECA/nicotine interaction. Third, compared with the immunohistochemistry technique employed in this study, the use of semiquantitative methods for protein analysis such as Western blots provides a better assessment of protein expression. Nevertheless, Western blots on NTS neuronal tissues suffer two drawbacks. The NTS is a tiny neuroanatomical area of the dorsomedial medulla oblongata measuring approximately 300–800 µm in adult rat brains, which necessitates the use of some enhancing procedures to amplify measured proteins [80]. Moreover, the possibility must be considered that micropunches of tissues taken from the NTS could be contaminated with nearby non-NTS brain tissue and produce inaccurate results. In this regard, one of the major advantages of immunohistochemistry is the specificity and accuracy of staining proteins localized in the NTS area.

## 4. Materials and Methods

### 4.1. Animals

The experimental animals employed in this study were adult male Wistar rats (220–250 g, animal Facility, Faculty of Pharmacy, Alexandria University). Rats were kept at an ambient temperature and had free access to standard rat chow and water. A total of 96 rats were used in this study. The sample size calculation was performed based on power analysis using G*Power 3.1.9.7 software [81]. Experiments were performed in accordance with the ARRIVE guidelines and the faculty guidelines for the use of experimental animals and were approved by the Animal Care and Use Committee of the Faculty of Pharmacy, Alexandria University (AU/06.2020.6.7.2.73).

### 4.2. Drugs

Nicotine (Merck Schuchardt OHG, Hohenbrunn, Germany), infliximab (Remicade^®^, 100 mg vial, Janssen Biotech, Inc, Horsham, PA, USA), VUF5574 (N-(2-Methoxyphenyl)-N’-[2-(3-pyrindinyl)-4-quinazolinyl]-urea), SP 600125 (1,9-Pyrazoloanthrone), Wortmannin, IB-MECA (N(6)-(3-iodobenzyl)-5′-N-methylcarboxamidoadenosine), PD 98059 (2-(2-Amino-3-methoxyphenyl)-4H-1-benzopyran-4-one), (Sigma Chemical Co., St. Louis, MO, USA), benzathine benzyl penicillin (Pencitard^®^ 1,200,000 I.U, ACDIMA, Cairo, Egypt), heparin sodium (Heparin Sodium^®^ 5000 I.U/mL, El-Nile Co., Cairo, Egypt), povidone iodine solution (Betadine^®^ 10%, El-Nile Co., Cairo, Egypt), thiopental sodium (Thiopental^®^ 500 mg vial, EPICO, Alexandria, Egypt).

### 4.3. Cecal Ligation and Puncture (CLP)

This procedure was used for the induction of sepsis as described by others [13,82]. One day before the cardiovascular measurements, the rats were anesthetized with thiopental (50 mg/kg, i.p.), a midline laparotomy of nearly 1.5 cm was carried out, and the cecum was exposed. Approximately one third of the distal end of the cecum was ligated using surgical sutures. Three punctures were performed on the same side of the cecum using a 21-gauge needle, then the cecum was gently squeezed to allow the extrusion of the fecal matter into the peritoneal cavity. Finally, the cecum was returned to the peritoneal cavity, and the abdominal musculature and skin were stitched.

### 4.4. Intracisternal Cannulation

This procedure was conducted 4 days before intravascular cannulation (i.e., 5 days before the experimentation day), as illustrated earlier [83]. After the induction of anesthesia with i.p. thiopental (50 mg/kg), the rat head was fixed in a David Kopf stereotaxic frame (David Kopf instrumentation, CA, USA). A guide cannula of 23 G (Small Parts, Miami, FL, USA) was passed between the occipital bone and cerebellum to be inserted into the cisterna magna. A dental acrylic luting cement (Glass Ionomer, Shanghai, China) was applied to secure the position of the guide cannula. Each rat was injected i.m. with 60,000 U of benzathine benzyl penicillin antibiotic and left to recover from anesthesia. Daily monitoring of the rats revealed no unexpected adverse events.

### 4.5. Intravascular Cannulation

Intravascular cannulation was performed [83] right after CLP or the sham operation. In thiopental (50 mg/kg, i.p.)-anesthetized rats, polyethylene catheters were introduced into the femoral vessels, the vein for i.v. administration of drugs and the artery for hemodynamic monitoring, respectively. Utmost attention was taken to ensure the integrity of the sciatic nerve to avoid movement disability in rats during experimentation. Thereafter, tunneling of catheters subcutaneously and exteriorization at the back of neck were performed cautiously, followed by flushing with heparinized saline (100 U/mL) and plugging with stainless steel pins. Experiments were performed 24 h later after connecting the arterial line to a BP transducer (model P23XL; Astro-Med, West Warwick, RI) that was connected via a MLAC11 Grass adapter cable to a computerized data acquisition system fitted with LabChart-7 pro software (Power Lab 4/35, model ML866/P; AD Instruments Pty Ltd., Castle Hill, Australia).

### 4.6. BRS Measurement

Baroreflex sensitivity (BRS) was determined using the vasoactive method [36,84]. According to this method, decrements and increments in HR in response to increases and decreases in BP elicited by bolus i.v. injections of randomized doses (1–16 μg/kg) of PE or SNP, respectively. The injection volume of each dose was 0.1 mL/200 g body weight, and a 5-min interval was allowed between each two successive doses. Changes in mean arterial pressure (MAP) and HR, from baseline before injection, were calculated. Baroreflex curves for PE and SNP were constructed by plotting changes in HR against the respective changes in MAP. BRS was then assessed by computing the slopes of the regression lines of the baroreflex curves (BRS-PE and BRS-SNP).

### 4.7. Immunohistochemistry

The immunohistochemical expression of NFκB and NOX2 was determined in the nucleus tractus solitarius (NTS), as described in previous reports [85]. The rat brainstem was fixed in formalin (10%) and embedded in blocks of paraffin. About 5-μm sections of rat brainstem NTS (−12.0 mm to −12.48 mm relative to bregma) [86] were sliced using a rotary microtome (Leica Biosystems, Nussloch, Germany), placed on positively charged adhesion glass slides (Epredia™, Braunschweig, Germany), deparaffinized in xylene and rehydrated in a series of descending concentrations of ethyl alcohol (100, 95 and 70%). For heat-induced epitope retrieval, the slides were immersed in 10 mM citrate buffer solution and incubated in a microwave at power 100 for 1 min then power 30 for 9 min. The slides were left to set to room temperature, followed by the application of 3% hydrogen peroxide for 10 min to block the endogenous peroxidases. The primary polyclonal antibodies against NFκB p65 (1:300, rabbit anti-NFκB p65, Bioss TM, USA) and NOX2 (1:250, rabbit anti-NOX2, ThermoFisher, Waltham, MA, USA) were applied to the slides and incubated overnight at 4 °C. On the following day, the secondary antibody (Horseradish peroxidase conjugate, Dako Agilent^®^, Santa Clara, CA, USA) was applied for 30 min followed by the chromogen 3,3′-diaminobenzidine (Dako Agilent^®^, Santa Clara, CA, USA). Between-steps washing was performed using a diluted washing buffer (Dako Agilent^®^, Santa Clara, CA, USA). Finally, sections were counterstained with hematoxylin and immersed in ascending concentrations of alcohol and xylene. For each section, approximately 10 images were taken using an Optika^®^ Optikam B9 digital camera mounted on an Optika^®^ B-193 microscope using the company’s Vision Lite software version 2.13. The Fiji ImageJ software version 1.51n (National Institutes of Health, Bethesda, MD, USA) was used to quantitate the immunohistochemical signal using the color deconvolution plugin. The intensity of the NTS brown color that exceeded a cut-off threshold was expressed as an area percentage and taken to reflect the immunohistochemical expression of targeted proteins.

### 4.8. Protocols and Experimental Design

#### 4.8.1. Modulation by Central A3ARs of Cholinergic Amelioration of CLP-Induced Neuroinflammation and Baroreflex Dysfunction

**Figure 7:**
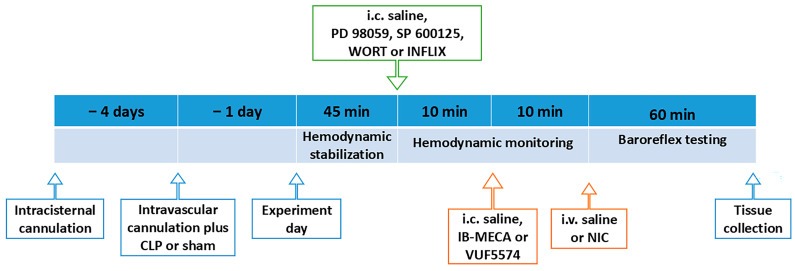
The timeline of surgical procedures and drug regimens employed in experiments.

Figure 7 depicts the surgical procedures and drug regimens employed in the experiments. On the experiment day (24-h following intravascular cannulation and sham or CLP), the arterial catheter was connected to a pressure transducer for measurement of BP and HR, as mentioned earlier. After a stabilization period of at least 45 min, rats were assigned to 8 treatment groups (*n* = 8 each): sham/saline (i.v.), CLP/saline (i.v.), CLP/nicotine (25 μg/kg, i.v.) [18], CLP/nicotine (100 μg/kg, i.v.) [18], CLP/IB-MECA (selective A3AR agonist, 4 µg/rat, i.c.) [87], CLP/IB-MECA (i.c.)/nicotine (100 μg/kg, i.v.), CLP/VUF5574 (A3AR antagonist, 2 µg/rat, i.c.) [36], and CLP/VUF5574 (i.c.)/nicotine (100 μg/kg, i.v.). These treatments were randomly assigned to the respective rat groups using the simple randomization sequence, which is based on a single sequence of random assignments. Baroreflex curves for PE and SNP were constructed as described above. Rats were excluded from the experiment if arterial or venous catheters were blocked or disconnected. At the conclusion of the experiment, rats were euthanized with an overdose of thiopental (100 mg/kg), and the brainstem was excised, fixed in 10% formaldehyde, and processed for immunohistochemical studies, as detailed above. Blinding could not be adopted as surgical procedures and drug administration protocols were implemented by the same researcher.

#### 4.8.2. Modulation by Central PI3K/MAPK/TNFα Signaling of A3AR-Nicotinic Baroreflex Interaction

Because data from the previous experiment revealed that A3AR activation by IB-MECA blunted the nicotinic counteraction of baroreflex dysfunction induced by sepsis, we investigated the effect of central inhibition of separate constituents of the PI3K/MAPK/TNF-α cascade on the IB-MECA/nicotine interaction. Four groups of conscious CLP rats (n = 8 each) were randomly assigned to receive one of the following regimens: (1) PD 98059 (MAPK-ERK inhibitor, 10 µg/rat, i.c.) [63,64]/IB-MECA (4 µg/rat, i.c.)/nicotine (100 μg/kg, i.v.), (2) SP 600125 (MAPK-JNK inhibitor, 30 µg/rat, i.c.) [63,64]/IB-MECA (4 µg/rat, i.c.)/nicotine (100 μg/kg. i.v.) (3) wortmannin (PI3K inhibitor, 0.5 µg/rat, i.c.) [63,64]/IB-MECA (4 µg/rat, i.c.)/nicotine (100 μg/kg, i.v.) or (4) infliximab (TNF-α inhibitor, 100 µg/rat, i.c.) [88,89]/IB-MECA (4 µg/rat, i.c.)/nicotine (100 μg/kg, i.c.). Ten-min intervals were left between sequential drugs in each regimen and BRS was assessed using the vasoactive method as described above. Rats were excluded from the experiment if arterial or venous catheters were blocked or disconnected. Rats were euthanized thereafter with an overdose of thiopental (100 mg/kg). The brainstem was removed, fixed in 10% formaldehyde, and processed for measurement of NFκB and NOX2 expressions in NTS using immunohistochemistry.

### 4.9. Statistical Analysis

The sample size was calculated using power analysis by G*Power 3.1.9.7 software [81]. Values are expressed as means ± SEM. The relation between changes in MAP and associated changes in HR was assessed using regression analysis for individual animals, as previously described [36]. The slope of the regression line (regression coefficient) expressed as beats/min/mmHg was computed and taken as an index of BRS. The one-way ANOVA followed by the Tukey’s post-hoc test was used to test for significance. The GraphPad InStat software release 3.05 was used for the analysis and probability levels of less than 0.05 were considered significant.

## 5. Conclusions

This study demonstrates that systemically administered nicotine elicited a dose-dependent reversal of the depressed reflex bradycardic activity seen in septic rats. Further agonist and antagonist studies highlight an essential role for functional central A3ARs in triggering the depressant effect of sepsis on baroreflex heart rate control and counteracting the rectifying effect of nicotine on baroreflex function in sepsis. At the molecular level, A3AR/cholinergic interaction on baroreflexes appears to be principally modulated by central PI3K/MAPK-JNK/NFκB/TNFα signaling. Therapeutically, this study highlights the importance of pharmacological elimination of A3ARs as a potential therapeutic strategy for improving cardiovascular dysfunction provoked by the septic insult. Clinical studies are certainly needed to reaffirm this view.

## Data Availability

Raw data have been uploaded as Supplementary Files.

## Supplementary Materials

The following supporting information can be downloaded at: https://www.mdpi.com/article/10.3390/ph18030388/s1, Table S1: BRS-sham-CLP-NIC; Table S2: BRS-A3; Table S3: BRS-A3-MAPKs; Table S4: A3-NFkB expression (brainstem); Table S5: A3-NOX2 expression (brainstem).

## Abbreviations

The following abbreviations are used in this manuscript:

BP	Blood pressure
BRS	Baroreflex sensitivity
CLP	Cecal ligation and puncture
ERK	Extracellular signal-regulated kinase
HR	Heart rate
JNK	c-Jun N-terminal Kinase
MAP	Mitogen-activated protein kinase
NFκB	Nuclear factor kappa B
NOX2	Nicotinamide adenine dinucleotide phosphate (NADPH) oxidases 2
NTS	Nucleus tractus solitarius
PE	Phenylephrine
PI3K	Phosphoinositide-3 kinases
SNP	Sodium nitroprusside
TNF-α	TNF-α Tumor necrosis factor-α
